# How to assess the reliability of cerebral microbleed rating?

**DOI:** 10.3389/fnagi.2013.00057

**Published:** 2013-09-26

**Authors:** Hugo J. Kuijf, Susanne J. van Veluw, Max A. Viergever, Koen L. Vincken, Geert Jan Biessels

**Affiliations:** ^1^Image Sciences Institute, University Medical Center UtrechtUtrecht, Netherlands; ^2^Department of Neurology, Brain Center Rudolf Magnus, University Medical Center UtrechtUtrecht, Netherlands

**Keywords:** inter-rater reliability, Cohen's kappa coefficient, ICC, Dice's similarity coefficient, cerebrovascular diseases, microbleeds, MRI imaging

Interest in cerebral microbleeds has grown rapidly over the past years. The need for sensitive and reliable detection of microbleeds has spurred the development of new MR sequences and standardized visual rating scales (Cordonnier et al., 2009; Gregoire et al., 2009). The value of these rating scales is currently assessed by measuring the inter-rater agreement, which is commonly determined using Cohen's kappa coefficient (κ) or the intraclass correlation coefficient (ICC). With the recent increase of MR scanner field strength to 3T and even 7T, the sensitivity of microbleed detection has grown significantly, whence often multiple microbleeds are found in a single subject (Brundel et al., 2012; de Bresser et al., 2012). As a result of this, researchers no longer solely focus on the absence or presence of microbleeds, but aim at determining their exact count and location as well.

Our concern is that, with this shift of focus, the measures that are in use to validate the reliability of microbleed ratings are no longer up-to-date. If the interest is confined to the presence or absence of microbleeds, the inter-rater agreement can be adequately assessed using κ. However, with multiple microbleeds in an individual subject, determining the inter-rater agreement using a measure that does not consider the number and location of the microbleeds appears inadequate. In other words, raters who agree on the presence or absence of microbleeds in an individual subject might disagree on their count or distribution.

The fact that κ might be an unsuitable measure for studies that are interested in microbleed count has not gone unnoticed. Recently, more studies are reporting the ICC as a measure for inter-rater agreement. This measure partly solves the aforementioned problem, because it takes the number of microbleeds into account. However, microbleed location is not taken into account in determining the ICC. Two raters might agree on the same microbleed count, while having counted different microbleeds. A second important drawback of the ICC is that it is data-dependent. An outlier subject that has many more microbleeds than the other subjects (e.g., a count of > 100 when the median is 2), will highly influence the ICC. This will thwart reliable determination of the inter-rater agreement that does not change because of an outlier subject. This is illustrated in Example A.

## Example A

In this example, the influence of an outlier subject on the determined inter-rater agreement using ICC is demonstrated. A group of 45 subjects (18 with early Alzheimer's disease and 27 controls) was recruited from a consecutive series of patients referred to our hospital. All subjects underwent a 7T MRI acquisition with, amongst others, a 3 D dual-echo gradient echo weighted sequence (see Brundel et al., 2012 for details). Written informed consent was given by all subjects and the study was approved by the institutional review board. Presence, count, and exact location of microbleeds was assessed by two independent human raters. Based on these ratings, the average number of microbleeds per subject was four, the median was one, and the ICC = 0.91. Among these subjects, there was an outlier subject who had eighty microbleeds. If this subject was excluded, the average number of microbleeds dropped to two, the median was still one, and the ICC decreased to 0.41. This decrease cannot be attributed to a variation in performance of the raters, but is solely caused by the in- or exclusion of the outlier subject. This is further supported by simulations, available to the reader on: http://www.isi.uu.nl/People/Hugok/microbleeds/simulator/

The data-dependency of the ICC is quite obvious in the given example. Nevertheless, a similar but more subtle effect will occur when newer, highly sensitive MR scanning techniques are used. As the prevalence and number of microbleeds increases (owing to more sensitive detection techniques), the ICC increases as well, even when all ratings are performed by the same raters. This is demonstrated in Example B.

## Example B

**Figure 1:**
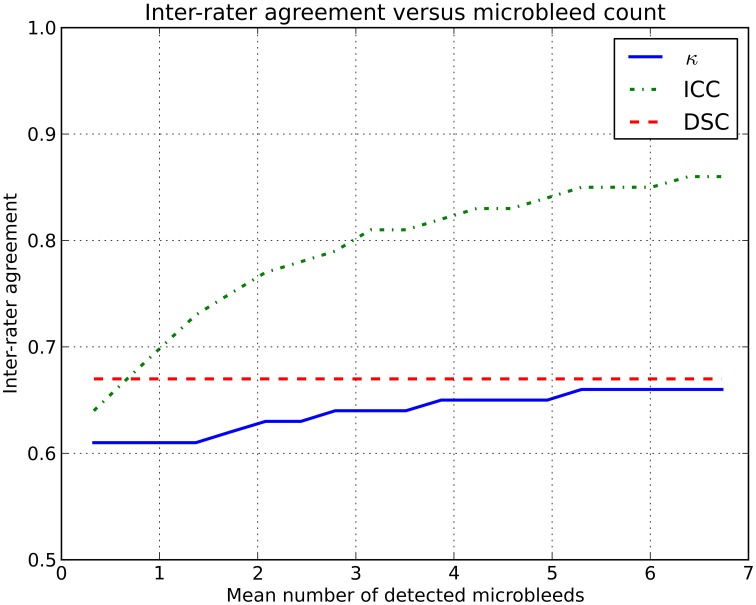
**Inter-rater agreement in simulations where two raters (with fixed sensitivities of 75 and 60%) rate microbleeds in different datasets with an increasing number of microbleeds**.

Using the simulator, two (virtual) human raters were simulated. Microbleed detection by these raters was simulated in two groups of subjects with different microbleed prevalence. The prevalence of microbleeds in the first group of subjects (*L*) was low, with on average 0.2 microbleeds/subject and a prevalence of 19%. The second group of subjects (*H*) had on average 3.4 microbleeds/subject with a prevalence of 84%. The two (virtual) human raters (*X* and *Y*) had a fixed sensitivity for microbleed detection of 75 and 60%, respectively. The intra-rater agreement between *X* and *Y* on *L* was ICC = 0.64 and on *H* was ICC = 0.81. More data are shown in Figure 1.

This rise in inter-rater agreement as expressed by the ICC is solely caused by the increase in microbleed count, since all other factors were stable or eliminated in the simulations. This clearly shows the data dependency of the ICC (or κ).

To overcome these issues, we propose the use of a similarity index, notably the Dice similarity coefficient (DSC), as a more reliable measure of inter-rater agreement (Dice, 1945). This measure could be reported in studies rating microbleeds, or other pathologies, to complement existing measures as κ or ICC. The DSC is computed according to the following formula: $\text{DSC}=\frac{2\left|X\cap Y\right|}{\left|X\right|+\left|Y\right|},$ where *X* and *Y* are the microbleeds rated by each of the two raters individually. The set |X ∩ Y| contains the microbleeds that are identified by both raters at the exact same location, i.e., the overlap. For example: a subject has five microbleeds, of which three were identified by rater *X* and four by rater *Y*. Two microbleeds identified by rater *X* were also identified by rater *Y*. The resulting inter-rater agreement $\text{DSC}=\;\frac{2^{*}2}{3+4}=0.6.$ If the DSC is computed for Examples A and B, the inter-rater agreement does not change because of outlier subjects or an increase in prevalence (see also Figure 1 and the online simulator).

The DSC has added value, because it considers the agreement of detection for every single microbleed, regardless if it occurs in a subject with a single microbleed or many microbleeds. If two raters annotate a microbleed in a subject, they will only reach agreement if this is the exact same microbleed on the exact same location in the brain. This removes the influence of outlier subjects and provides a more direct reflection of the performance of raters in microbleed detection. Computation of the DSC should not require additional time as compared to computing κ or ICC. The verification that two raters identified the same microbleed is already standard procedure during a consensus meeting, thus this should simply be noted to compute the DSC.

The simulated effect demonstrated in Figure 1 is also present in real data, albeit more subtle. As MR field strength increases and more microbleeds are detected, thus increasing κ /ICC, the rating of microbleeds becomes more difficult, thus decreasing the κ /ICC. However, this difficulty of rating microbleeds at high resolution images is not expressed to its full extent, as the inter-rater agreement is artificially high owing to the used inter-rater agreement measure.

The DSC has more advantages over existing measures for inter-rater agreement. With the increasing number of studies performing microbleed rating, there is also an increasing request for (semi-) automated microbleed detection techniques (Kuijf et al., 2013). To compare these techniques with human raters, measures as sensitivity are reported, with a reference (“ground truth”) defined by one or multiple human raters. Alternatively, the DSC may be used as a goodness-of-agreement measure. When the DSC is also used for inter-rater agreement, a direct and unbiased comparison can be made between rater performance and the performance of automated techniques. Furthermore, when training novice raters for microbleed detection on scans acquired with high MR field strength, the use of the DSC may indicate the performance of a rater compared with an established ground truth.

In conclusion, we have given arguments why the DSC provides a good measure of inter-rater agreement in studies that aim at determining cerebral microbleed count and locations. The ICC and κ are valid measures of rater agreement for detecting absence or presence of microbleeds in a subject, but become inaccurate in studies where subjects have multiple microbleeds, as is typically true for high-field (3T, 7T) brain MRI acquisitions.
